# Impacts of COVID-19 on Food Choices and Eating Behavior among New Zealand University Students

**DOI:** 10.3390/foods13060889

**Published:** 2024-03-15

**Authors:** Jessica C. McCormack, Mei Peng

**Affiliations:** 1 Sensory Neuroscience and Nutrition Lab, Department of Food Science, University of Otago, Dunedin 9016, New Zealand; jessica.mccormack@otago.ac.nz; 2 Riddet Institute, Palmerston North 4410, New Zealand

**Keywords:** COVID-19, food choices, nutrition, diet, post-COVID, sensory dysfunction

## Abstract

Extensive research suggests that COVID-19 infection can lead to persistent changes in taste and smell perception. These sensory changes have the potential to exert lasting impacts on dietary choices, nutrition, and body weight. This study aims to explore COVID-related shifts in dietary intake among New Zealand university students. We conducted a survey involving 340 university students who had experienced COVID-19 infection between 2022 and 2023. Participants reported any changes in eating behavior since before the pandemic and were asked to complete a 24-h food record. Participants’ total daily energy intake, macronutrient intake, and composition were then compared with data collected from a similar cohort before the COVID-19 pandemic, spanning from 2017 to 2019. Dietary outcomes were compared using MANCOVA while controlling for individual age, gender, ethnicity, and BMI. Approximately 25% of participants reported experiencing sensory alterations with COVID-19, with those participants more likely to report changes in their experience of sweet tastes but not salty or fatty foods. Analyses of the pre- and post-COVID cohorts revealed that participants in the post-COVID group exhibited significantly higher consumption of protein and sodium. Understanding the long-term impact of COVID-19 infection may offer crucial insights into the role of chemosensory perception in dietary behavior.

## 1. Introduction

Research has suggested that many individuals who are infected with SARS-CoV-2, the virus responsible for the COVID-19 global pandemic, experience sensory symptoms, such as loss or distortion of smell or taste abilities [1,2,3,4,5,6]. Given that sensory perception plays a significant role in influencing food preference and selection, sensory alterations can directly impact individual energy and macronutrient intake, as well as diet quality [7,8,9,10]. However, the effects of COVID-19 on dietary choices remain unclear. Importantly, conducting cross-sectional or case-control studies comparing exposed and unexposed individuals is challenging due to surges in COVID-19 infections and limited access to polymerase chain reaction (PCR) testing to confirm infection. To circumvent these challenges, the current study uses a retrospective cohort design to compare eating behavior in university students to pre-existing data collected prior to the COVID-19 pandemic.

### 1.1. Sensory Impact of COVID-19

Relatively early in the pandemic, changes in smell and taste were identified as distinctive symptoms of COVID-19 with potential diagnostic value [4,11]. Meta-analyses have found that sensory changes were highly prevalent in 2020 and 2021, with as many as 40–60% of people reporting changes in smell or taste [1,12] and 20% of patients reporting the onset of smell and taste changes without other symptoms [1]. Studies also suggest, however, that the prevalence of sensory symptoms may vary by region [13] and may be less common in the more recent COVID-19 variants (e.g., Omicron) [14]. For example, only 11% of Australian COVID-19 patients reported sensory changes compared to 55% of European COVID-19 patients [13]. A study comparing chemosensory dysfunction in a cohort of patients infected in January and February 2022 to those infected in March and April 2020 found that self-reported chemosensory dysfunction was significantly lower in those infected in 2022 [15]; less than 25% of those infected in early 2022 reported altered sense of smell compared to more than 60% in early 2020.

Although most patients recover their sense of smell and taste within 3 months of infection [16], studies have reported the long-term impact of COVID-19 on smell and taste for some patients even after they have recovered from COVID-19. For example, a study recruiting patients with symptoms of smell distortion found that 83% had long-term distortion of smell related to COVID-19 [17]. A recent meta-analysis reported persistent self-reported smell and taste loss in 5.6% and 4.4% of patients, respectively [18]. Long-term dysfunction of taste and smell can have a significant impact on quality of life, including reduced appetite and enjoyment of food, concern about body odors, and risk of injury [19].

### 1.2. Changes in Food Choice and Diet due to COVID-19

Several changes in eating habits and dietary behaviors have been reported in relation to the COVID-19 pandemic, although they may appear to be contradictory. These changes include increases in snacking behaviors and comfort eating [20,21], increased adherence to a healthy diet [22], increased consumption of high energy density foods [23,24,25], increased home cooking [22,26], and increased alcohol consumption [27]. Negative changes in dietary behavior—such as snacking and alcohol consumption—were associated with increased weight gain and reduced physical activity [21].

However, there is limited research on direct links between COVID-related sensory symptoms and eating behavior, and these reported changes may not be directly attributable to COVID-19 exposure. To date, there have been few empirical studies specifically evaluating possible shifts in eating behavior due to COVID-19 infection. A systematic review of dietary behaviors in people who experienced chemosensory dysfunction following COVID-19 found only five quantitative studies meeting the criteria [28]. However, the studies were primarily cross-sectional studies where participants self-reported changes in food-related behavior. For example, a study involving Danish adults in the post-acute phase of COVID-19 found that participants reported reducing the size of the meals post-infection and reducing consumption of animal products compared to before COVID-19 infection [29]. Other studies have compared measures of eating behavior before and after COVID-19, but not specifically in those who were exposed to COVID-19 infection, with a number of studies focusing on changes in eating behavior before and after lockdowns [30,31,32,33]. Many factors may have influenced dietary changes over the last 3 years, including supply chain disruptions, COVID-19 lockdowns, increasing awareness of climate change, and rising food prices due to inflation. It remains unclear whether the combination of sensory changes and lifestyles interruptions during COVID-19 has been the primary driver of long-term dietary changes.

### 1.3. New Zealand Exposure to COVID-19

Due to public health measures, including strict national and regional lockdowns and travel restrictions, New Zealand was able to eliminate the spread of COVID-19 in the community in 2020 and had relatively low exposure to COVID-19 up until late 2021 [34,35]. The change from an elimination strategy to a minimization and protection strategy in December 2021 [36] saw cases of COVID-19 infection in New Zealand surge from early February 2022, peaking at 22,025 new daily infections on 6 March 2022 [37]. During the early phase of the pandemic, strict isolation policies were in place, with households required to isolate for 14 days from a positive test result. This was reduced to 7 days in April 2022, and the requirement for household contacts to isolate was removed, and as of August 2023, isolation was no longer mandatory [38].

Given the strict public health measures employed in New Zealand, previous studies of the effect of COVID-19 on dietary patterns in New Zealand have focused on the lockdown periods when retail stores were limited in their operation [39,40]. Only one previous study looked at changes in dietary intake, specifically fruit and vegetable intake, from the period before COVID-19 to after COVID-19. However, the study was conducted on secondary school students, and participants were not selected based on their COVID-19 exposure. University students represent a convenience sample of relatively healthy participants with a lower risk of long-term health conditions affected by COVID-19 infection.

### 1.4. Objective

This study aims to explore changes in eating behavior related to COVID-19 infection and test for differences in dietary intake and composition among university students before and after the COVID-19 pandemic.

In this project, we conducted a survey of university students’ dietary behavior, including food choices and intake and post-COVID-19 infection. We compared participants who were infected with COVID-19 to pre-existing data collected by the Sensory Neuroscience Lab between July 2017 and July 2019, which used the same measures and collected data from a similar cohort of university students living in a similar food environment.

## 2. Materials and Methods

### 2.1. Study Design

We used a retrospective cohort design to compare a cohort of university students with post-COVID-19 infection and pre-existing cohort data collected before the COVID-19 pandemic. The research was approved by the University of Otago Ethics Committee (22/097), and the protocol was pre-published (https://doi.org/10.1101/2022.09.28.22280475, accessed on 15 March 2024).

### 2.2. Setting

Participants were primarily drawn from the student cohort at the University of Otago, Dunedin, between September 2022 and August 2023. At the University of Otago, exposure to COVID-19 remained low for most of 2020 and 2021, with only 234 cases of COVID-19 in 2021 [41]. As of February 2024, there have now been 190,424 cases of COVID-19 reported in the Otago region [42], representing more than half the population of the region. This number is likely underestimated because of a decline in the declaration of COVID-19 since 2023.

### 2.3. Participants

Participants were eligible to participate if they met the following criteria:

- were aged 18 or older;
- were a university student;
- were generally healthy (i.e., not on regular medication);
- had been infected with COVID-19;
- had COVID-19 infection was confirmed at the time by PCR test or rapid-antigen test (RAT);
- could give e-consent.

Participants were excluded if they were under the age of 18, taking regular medication to manage a physical condition, had chronic sensory dysfunction, had been diagnosed with an eating disorder in the last 2 years, or were currently following a weight-loss or weight-gain dietary program.

### 2.4. Sample Size

The sample size calculation was based on changes in energy intake in university students before and during the COVID-19 pandemic reported in previous studies [24,39,40]. Based on a mean effect size of 0.22, in order to detect a significant difference in energy intake (*p* < 0.05) with 80% power, we recruited a sample of 326 participants exposed to COVID-19 infection.

### 2.5. Data Collection

Participants completed an online survey via REDCap electronic data capture hosted by the University of Otago [43]. Baseline demographics included year of birth, ethnicity, gender, year of study, and socioeconomic information, along with health-related information such as diet, antidepressant use, and smoking or vape use. Participants were asked to report on their experience with COVID-19 infection, including the timing of infection and their vaccination status and location at the time of infection. Participants were asked if they experienced any changes in taste or smell in their most recent infection. If they answered ‘yes’, they indicated whether they experienced a complete loss of smell or taste or partial loss of taste and could provide details of how their taste or smell changed. Participants were also asked how long it took for sensory perception to return to normal. All participants were asked to report on perceived changes in the taste of sweet, salty, and fatty foods and rated the taste as weaker, stronger, or having no change during their infection. These properties were selected as they were the most likely to be affected based on previous studies of patients with smell loss [44]. Participants used a five-point Likert scale (from much more to much less) to report on changes in food choices and eating behavior since before the pandemic, using food categories such as sweet and salty snack foods, fast food, meal preparation kits, baking, and meals prepared from scratch based on previous studies looking at changes in purchasing patterns [21,24,45]. The full survey is included in Supplementary Materials S1. Participants also completed the Dutch Eating Behavior Questionnaire (DEBQ) [46] as a measure of eating behavior.

After completing the online survey, participants were invited to attend a drop-in session where the researchers measured their height and weight and gave them verbal instructions on how to complete a 24-h weighed food record [47]. The food record was adapted for New Zealand participants and reviewed by a registered dietician. Participants were asked to return their food records within 1 week of the drop-in session, and 2 reminders were sent after 1 week.

#### Datasets

Pre-COVID data were collected between July 2017 and July 2019 as part of research projects conducted at the Sensory Neuroscience and Nutrition Lab, University of Otago. All participants had given consent to use their data for future research activities. Ethical approval for these studies was obtained from the University of Otago Ethics Committee. Although these data came from multiple studies within the specified time period, all studies drew from the same population (i.e., university students), and the general inclusion and exclusion criteria were consistent across studies. All studies included the DEBQ and measured food intake using a 24-h or 4-day weighted food record. Where a 4-day food record had been collected, only the first day of the food record was included for analysis. The collected demographic information included age, gender, ethnicity, and BMI.

The post-COVID data consisted of all food records returned from participants recruited to the study, along with data collected in early 2022 of participants who had been infected with COVID-19 prior to the time of data collection. Participants in the additional dataset completed a 4-day weighed food record, and demographic information obtained included age, gender, ethnicity, and BMI.

### 2.6. Data Analysis

#### 2.6.1. Data Extraction

Responses from food records were transferred into daily energy intake (in kJ) using FoodWorks (Xyris Software 10, Brisbane, QLD, Australia). Along with daily intake, we also extracted specific macronutrient intake (fat, carbohydrate, protein), sugar, and sodium intake. We excluded participants from the dietary intake analysis who were missing their food records or had an incomplete or implausible food record.

#### 2.6.2. Statistical Analysis

Statistical analysis was conducted using IBM SPSS Statistics 30.0 (IBM, Armonk, NY, USA) and RStudio (PBC, Boston, MA, USA). Baseline characteristics of participants were summarized for the pre-COVID cohort and post-COVID cohort using mean and standard deviation (SD) for continuous variables and frequencies and percentages for categorical variables. Within the post-COVID cohort, baseline characteristics were summarized according to the experience of sensory dysfunction during COVID-19 infection. We conducted t-tests and chi-square to compare baseline characteristics across the two cohorts.

Intake measures were compared across cohorts using MANCOVA and controlling for moderators such as gender, weight, and age. The following measures of energy intake were extracted from the food records: daily energy intake (kJ), fat intake (g), protein intake (g), carbohydrate intake (g), sugar intake (g), and sodium intake (mg), and the mean and standard error calculated for each outcome. Any missing covariates were imputed from the group mode or average. We also evaluated any differences in intake in the post-COVID cohort between those who reported sensory change and those who did not.

## 3. Results

### 3.1. Post-COVID Cohort

**Figure 1:**
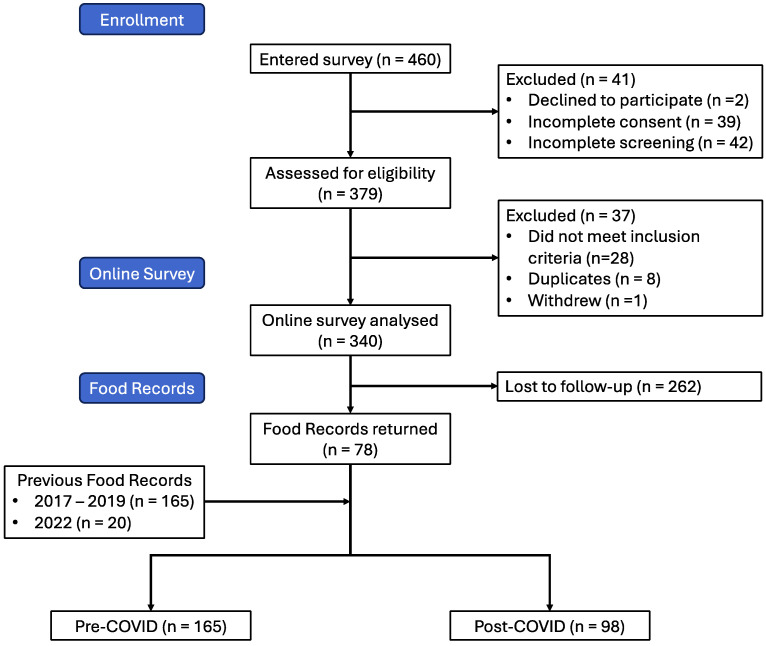
Flow diagram showing participant recruitment and retention through study.

A total of 460 responded to the study invitation, of which 41 declined to participate or did not complete the consent form and another 42 did not continue to the screening form. Of the 377 that completed the screening form, 28 were ineligible to participate. After excluding 8 duplicate participants and 1 participant withdrawal, we were left with a final sample of 340 participants in the post-COVID infection cohort (Figure 1).

The majority of participants were female (69.7%), and the mean age was 22 years old (Table 1). More than 70% of participants identified as New Zealand European, with 8.5% identifying as Māori and 4.4% identifying as Pacific. Most participants were in their third year or later (64.2%) and had been studying in Dunedin for more than 1 year. Few participants reported following a specific diet; plant-based was the most commonly reported diet. Participants generally reported being in good health (80.2%).

A total of 60% of participants reported only one infection of COVID-19, with 72.9% reporting their most recent infection in 2022. Less than 10% reported an infection before 2022. More than three-quarters were fully vaccinated (second dose or booster) at the time of infection. Most participants reported only mild severity, and this was consistent with the most common reported symptoms reported, which were sneezing (77.4%), sore throat (77.1%), and tiredness (77.9%). More severe symptoms, such as breathing difficulties and chest pain, were reported by less than 20% of participants. Sensory symptoms were reported by 25% of participants.

#### 3.1.1. Experience of Sensory Changes

One-quarter of participants (*n* = 85) reported that they experienced some kind of sensory change at the time of infection. The most commonly reported changes were a reduction in sense of taste (63.5%) and a reduction in smell (60.0%), with complete loss of taste or complete loss of smell reported by less than 25% of those reporting sensory changes. The vast majority reported that their sense of smell returned to normal within a week of their first symptom. However, four participants reported that their sense of smell or taste had not returned to normal after 6 months or longer.

We compared self-reported symptoms of COVID-19 and symptom severity across participants who experienced sensory changes and participants who reported no sensory changes. The two groups differed significantly in symptom severity (*p* < 0.001), with the proportion of participants reporting moderate and severe symptoms greater for those who experienced sensory changes. This was also apparent in the types of symptoms reported, with significantly more of those experiencing sensory changes reporting body aches, cough, tiredness, headache, nausea, breathing difficulties, and chest pain.

Participants were also asked if they experienced any changes in the intensity of sweet, salty, or fatty foods since infection with COVID-19. Overall, more than three-quarters of participants reported no change in the intensity of sweet, salty, and fatty foods. However, there was a significant difference between groups for sweet foods but not for fatty foods or salty foods. A greater proportion of participants who had sensory changes reported that sweet foods tasted weaker (32.9% vs. 3.9%).

#### 3.1.2. Impact of Sensory Changes on Food Intake

We analyzed the food records of 78 participants to compare food intake in participants who experienced sensory changes (*n* = 20) and those who did not report sensory changes. MANCOVA controlling for age, gender, and BMI groups suggested that the difference between these two sub-groups did not reach statistical significance (*F*(*6*, *68*) = 0.559, *p* = 0.0762, Wilks’ Λ = 0.953). Furthermore, there were no significant differences in sensory change for any of the measures of food intake (*p* > 0.05). Gender was the only significant covariate (*p* < 0.001).

### 3.2. Self-Reported Changes in Food Choices Post-COVID

**Figure 2:**
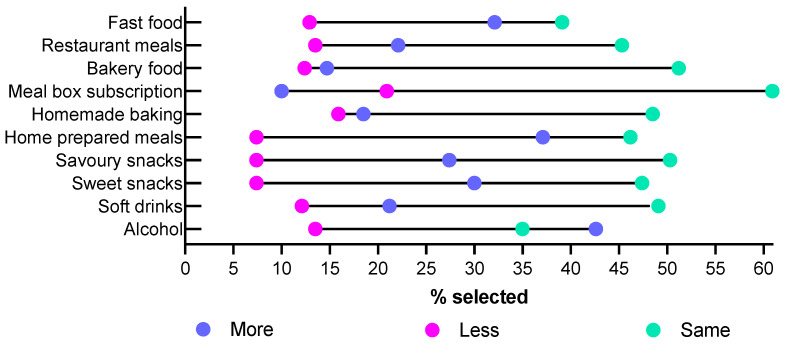
Self-reported changes in food purchasing behaviors since 2019 summarizing the percentage of participants who selected more or much more, less or much less, and no change/same.

Participants were asked to report on how often they purchased or prepared different foods compared to the same time in 2019 (Figure 2). For the majority of food purchasing behaviors, 45–60% of participants reported that there was no change in their behavior; only 35% of participants reported no change in their alcohol consumption, and 39% of participants reported no change in their fast-food consumption. More than a third of participants reported increases in purchasing behavior for alcohol and preparing meals from scratch, while only 10% reported an increase in the use of meal box subscriptions. At most, 15% of participants reported a decrease in behavior for any category, with only 7.4% reporting purchasing or consuming less sweet or savory snack foods or meals prepared from scratch.

### 3.3. Changes in Dietary Intake Post-COVID-19

We obtained 98 food records from participants who had recently had COVID-19 (78 24-h food records and 20 4-day weighed food records). Our pre-COVID group consisted of 165 food records collected between 2017 and 2019.

The two groups did not differ in gender, weight group, or ethnicity. However, participants in the post-COVID cohort were significantly younger than those in the pre-COVID cohort (Table 2). The groups also differed in their eating behavior according to the DEBQ, with higher average scores in restrained and emotional eating in the post-COVID cohort and higher average scores in external eating in the pre-COVID cohort.

We used MANCOVA to compare the effects of COVID-19 on food intake, taking into account the effects of covariates. Age, gender, ethnicity, and BMI groups were all included as covariates in the analysis.

**Figure 3:**
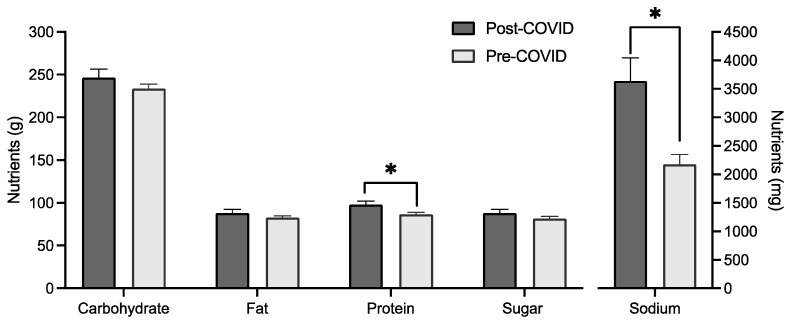
Macronutrient, sugar, and sodium (in mg) intake by cohort with mean and standard error. * indicates a significant difference between groups (*p* < 0.05).

MANCOVA analysis showed a significant difference in mean intake based on cohort when controlling for age, gender, and BMI groups (*F*(*6*, *252*) = 3.649, *p* = 0.002, Wilks’ Λ = 0.920). Between-subject effects found a significant difference for sodium intake (*F*(*1*, *257*) = 8.257, *p* = 0.004) and protein intake (*F*(*1*, *257*) = 12.315, *p* < 0.001), with mean protein and sodium intake higher for the post-COVID group (Figure 3). Age and gender were both significant covariates in the multivariate test.

## 4. Discussion

Our study of university students’ experiences of COVID-19 showed that most experienced relatively mild symptoms of COVID-19, and only 25% reported experiencing sensory changes, which were largely reduced smell and taste rather than complete loss. Participants who experienced sensory changes were more likely to report that the taste of sweet foods had changed but were not more likely to report changes in fatty or salty foods. Analysis of food records from participants infected with COVID-19 compared to food records from 2017 to 2019 found some changes in intake for protein and sodium. There was no difference between those who experienced sensory changes and those who did not.

Previous studies suggest that the quality of college students’ diets declined during COVID-19 lockdowns, with studies reporting decreased consumption of healthy foods (like fruits and vegetables) and increased consumption of confectionary products [48]. Our overall analysis found no difference in the total energy intake in our cohorts of university students before and after COVID, nor did we detect differences in energy intake from sugar per se. Intriguingly, there was a change in intake of protein and sodium, with the post-COVID group reporting increased consumption. Moreover, it is worth noting that, on average, both groups consumed more protein than the recommended daily intake (46 g per day for women and 64 g per day for men). Increased sodium intake is consistent with an increasing trend in sodium intake observed in large observation studies in the last decade. For example, a population survey in the United States of America reported significant increases in daily sodium intake from 1999 to 2016 [49]. Some studies have attempted to attribute the increased sodium intake to increased production and consumption of processed foods, in particular snacks [50], although more research is warranted to confirm such links.

A number of studies have found an association between micronutrients and trace elements and COVID-19 infection. For example, a study from Spain in 2020 found that low levels of zinc and vitamin A were associated with admission to intensive care units in patients with PCR-confirmed COVID-19 [51]. Similarly, a study of older COVID-19 patients in Switzerland found the majority of patients had micronutrient deficiencies, and the severity of COVID-19 disease progression was associated with levels of zinc, vitamin A, and folic acids [52]. Studies have suggested that deficiencies in micronutrients can impair immune function and, therefore, leave patients more susceptible to COVID-19 and increase the severity of symptoms and disease progression [53]. Deficiencies in micronutrients may also have an impact on appetite regulation [54] and, therefore, may relate to observed differences in macronutrient intake, although we did not observe any differences in overall intake.

Sensory changes were a relatively rare symptom reported by participants in our study. Approximately 25% of the participants reported sensory change, and only around 5% reported complete loss of taste or smell, compared to the 40–60% experiencing smell or taste changes in a meta-analysis of studies based on objective measures [4]. However, this finding is consistent with more recent evaluations of smell and taste loss in COVID-19 patients [55,56], with evidence suggesting that the occurrence of smell and taste changes has decreased in more recent variants of COVID-19 [57]. For example, an analysis of 12 reports of patients infected with COVID-19 during the Omicron wave had a pooled estimated olfactory dysfunction of only 13% [14]. Thus, this difference is most likely due to the prevalent strain at the time of infection. Specifically, previous studies concerned the Delta strain, which was the dominant variant in Europe in 2021, while most of our participants reported infections in 2022 when Omicron was the dominant variant. Similarly, most of our participants were fully vaccinated at the time of infection, which likely affected their experience of COVID-19 symptoms.

While relatively rare, there was a notable difference in the proportion of participants with self-reported sensory changes who reported changes in their perception of sweet foods but not fatty or salty foods, with almost a third reporting that sweet foods tasted weaker. Changes in the perception of sweet foods may result in increases in salty or fatty foods, with participants switching to salty or fatty snacks due to less enjoyment of sweet foods due to the dulled taste [7,58]. This is consistent with findings related to anosmia; for example, patients with clinical alteration in smell consistently showed a shift in preference toward savory and salty foods, and many reported reduced consumption of sweet foods and drinks [44]. Although this finding is self-reported, it is consistent with clinical testing of taste loss in COVID-19 patients compared to controls, which showed that sweet and bitter taste recognition was the most impaired in COVID-19 patients [3].

Similar to previous studies, self-reported changes in eating and purchasing behaviors were variable across participants [51]. A New Zealand study [59] conducted from March to April 2020, when New Zealand was in lockdown, found that preparing meals from scratch and preparing baked goods and bread increased for 40–60% of participants between 18 and 50 years old. We found similar levels of increased consumption of meals prepared from scratch (37% vs. 39%) but not for baking, where less than 20% of participants reported an increased consumption of homemade baked goods. The return of baked goods to baseline levels for most is not surprising as people have returned to in-person classes and work, which may constrain baking. The continued increase of at-home cooking may be a long-term effect of lockdowns and restricted access to fast food and restaurants during this period. During the acute phase of the pandemic, consumption of fast foods was reduced [60,61], but students in the present study reported that consumption of fast foods was more or about the same as pre-pandemic, which may be a result of the increased need for socialization after restrictions were removed. Consistent with other studies of changes in consumption patterns in university students, we found a self-reported increase in alcohol consumption [62,63].

### Strengths and Limitations

While COVID-related sensory changes are hypothesized to have impacts on eating behavior and dietary choices, it is challenging to perform controlled cohort studies due to the high prevalence of undetected infections. The temporal analyses provide a unique opportunity to test for COVID-related impacts on eating behavior. By using pre-existing data collected before the COVID-19 pandemic, we were able to compare similar cohorts of students pre- and post-COVID-19. However, a limitation of this study design is that we were unable to control for other changes that have occurred over the same time period, such as inflation and the increasing cost of food, changes in product formulation due to the increasing adoption of health star ratings [64], and the increasing adoption of plant-based and flexitarian diets [65]. Furthermore, although we used the same selection methods for both the pre-COVID and post-COVID populations, the analysis compares two different populations, which limits our ability to make one-to-one comparisons.

The study may have limited generalization to other countries due to the unique lockdown experiences of students living in New Zealand during COVID-19. Specifically, New Zealand had a much stricter lockdown than many other Western countries and maintained these restrictions for longer than other countries. The New Zealand experience was also unique in that New Zealanders had relatively low exposure to COVID-19 variants that had higher severity and hospitalization and, therefore, may have experienced less pronounced long-term effects of COVID-19 infection.

We obtained a high rate of responses for our online survey. However, the return of food records was relatively low, likely due to a higher participant burden to completing and returning physical food records compared to completing an online survey, which affected the study’s power. Due to the low rate of response, observed differences in dietary intake may be due to differences in the dietary patterns and activities of respondents compared to non-respondents. Another limitation is the use of food records to measure eating behavior, as opposed to lab-based measures. Food records, as self-report measures, are subject to biases in underreporting [66], while lab-based measures like the ad libitum buffet are free of such biases but less naturalistic. Furthermore, a study found that 24-h or 2-day weighed food records may be less accurate than longer food records [67].

## 5. Conclusions

Understanding the long-term impact of COVID-19 infection on dietary choices and nutrition is crucial for implementing effective public health measures in the post-COVID era. While our study suggests that COVID-19 may have lasting effects on diet choices, it is important to recognize that other changes in the food environment may also contribute to these changes. It is important for future research on these dietary changes to consider the impact of micro and macronutrients, as well as the wide range of changes that have occurred in our food environments since 2019. In future studies, it may be valuable to look at laboratory-based measures of food intake, such as ad libitum intake, and compare data across recent and past COVID-19 infections. Responding to COVID-19 and its potential impact on dietary choices represents an opportunity to narrow health disparities and better respond to the health needs of vulnerable communities.

## Figures and Tables

**Table 1:** Characteristics of post-COVID survey participants (n%).

		Overall	No Sensory Change	Sensory Change
		N = 340	N = 255	N = 85
Age: Mean (SD)		22.02 (3.83)	21.85 (3.49)	22.53 (4.70)
Gender				
	Female	237 (69.7)	171 (67.1)	66 (77.6)
	Male	93 (27.4)	77 (30.2)	16 (18.8)
	Other	6 (1.8)	4 (1.6)	2 (2.4)
Ethnicity				
	New Zealand European (NZE)	241 (70.9)	185 (72.5)	56 (65.9)
	Asian	50 (14.7)	35 (13.7)	15 (17.6)
	Māori	29 (8.5)	23 (9.0)	6 (7.1)
	Pacific	15 (4.4)	10 (3.9)	5 (5.9)
	Other	5 (1.5)	2 (0.8)	3 (3.5)
Year of study				
	First year	54 (15.3)	38 (14.9)	16 (18.8)
	Second year	63 (19.0)	49 (19.2)	14 (16.5)
	Third/final Year	160 (48.2)	122 (47.8)	38 (44.7)
	Post-grad	55 (16.6)	39 (15.3)	16 (18.8)
Current Household				
	Living alone	5 (1.5)	3 (1.2)	2 (2.4)
	Living with other adults	262 (77.1)	199 (78.0)	63 (74.1)
	Married/de facto couple	13 (3.8)	12 (4.7)	1 (1.2)
	Family with children	11 (3.2)	6 (2.4)	5 (5.9)
	Family with adults only	5 (1.5)	4 (1.6)	1 (1.2)
	Living in a residential college	43 (12.6)	31 (12.2)	12 (14.1)
	Other	1 (0.3)	0	1 (1.2)
2019 Household				
	Living alone	3 (0.9)	3 (1.2)	0
	Living with other adults	93 (27.4)	73 (28.6)	20 (23.5)
	Married/de facto couple	5 (1.5)	4 (1.6)	1 (1.2)
	Family with children	124 (36.5)	95 (37.3)	29 (34.1)
	Family with adults only	56 (16.5)	38 (14.9)	18 (21.2)
	Living in a residential college	30 (8.8)	24 (9.4)	6 (7.1)
	Other	19 (5.6)	11 (4.3)	8 (9.4)
Antidepressant use		23 (6.8)	17 (6.7)	6 (7.1)
Tobacco use				
	Smoker	16 (4.7)	13 (5.1)	3 (3.6)
	Vaper	110 (32.6)	71 (27.8)	29 (34.1)
	Non-smoker/non-vaper	226 (66.5)	172 (67.5)	54 (63.5)
Diet				
	No specific diet	288 (84.7)		
	Plant-based diet	27 (7.9)		
	Mediterranean diet	2 (0.6)		
	Low-carb diet	3 (0.9)		
	Other	9 (2.7)		
DEBQ				
	Restrained	2.53 (0.82)	2.54 (0.83)	2.50 (0.80)
	External	3.24 (0.55)	3.22 (0.55)	3.29 (0.56)
	Emotional	2.57 (0.78)	2.62 (0.77)	2.45 (0.78)

**Table 2:** Baseline demographics of post-COVID and pre-COVID cohort (*n*%).

		Pre-COVID	Post-COVID	*p*-Value
		*n* = 165	*n* = 98	
Age: Mean (SD)		29.37 (6.14)	22.88 (4.54)	<0.001
Gender				0.115
	Female	52 (31.5)	72 (73.5)	
	Male	113 (68.5)	23 (23.5)	
	Other	0	3 (3.1)	
Ethnicity				0.963
	NZE	115 (69.7)	70 (71.4)	
	Asian	28 (17.0)	21 (21.4)	
	Māori	6 (3.6)	2 (2.0)	
	Pacific	2 (1.2)	1 (1.0)	
	Other	14 (8.5)	4 (4.1)	
BMI: Mean (SD)			25.18 (5.08)	
Weight group				0.067
	Underweight	3 (1.3)	4 (4.1)	
	Normal weight	97 (58.8)	56 (57.1)	
	Overweight/obese	64 (38.8)	24 (24.5)	
DEBQ: Mean (SD)				
	Restrained	2.12 (0.72)	2.43 (0.90)	0.010
	Emotional	2.25 (0.81)	3.31 (0.58)	<0.001
	External	3.12 (0.49)	2.52 (0.84)	<0.001

## Data Availability

The original contributions presented in the study are included in the article/Supplementary Materials, further inquiries can be directed to the corresponding author.

## Supplementary Materials

The following supporting information can be downloaded at: https://www.mdpi.com/article/10.3390/foods13060889/s1, Supplementary Materials S1. Survey Questions.

## References

[1] Borsetto D., Hopkins C., Philips V., Obholzer R., Tirelli G., Polesel J., Boscolo-Rizzo P. (2020). Self-reported alteration of sense of smell or taste in patients with COVID-19: A systematic review and meta-analysis on 3563 patients. Rhinology.

[2] Burges Watson D.L., Campbell M., Hopkins C., Smith B., Kelly C., Deary V. (2021). Altered smell and taste: Anosmia, parosmia and the impact of long COVID-19. PLoS ONE.

[3] Cattaneo C., Pagliarini E., Mambrini S.P., Tortorici E., Mené R., Torlasco C., Perger E., Parati G., Bertoli S. (2022). Changes in smell and taste perception related to COVID-19 infection: A case–control study. Sci. Rep..

[4] Hannum M.E., Koch R.J., Ramirez V.A., Marks S.S., Toskala A.K., Herriman R.D., Lin C., Joseph P.V., Reed D.R. (2022). Taste loss as a distinct symptom of COVID-19: A systematic review and meta-analysis. Chem. Senses.

[5] Hannum M.E., Reed D.R. (2021). COVID-19-Associated Loss of Taste and Smell and the Implications for Sensory Nutrition. Sensory Science and Chronic Diseases: Clinical Implications and Disease Management, Joseph, P.V., Duffy, V.B., Eds..

[6] Gerkin R.C., Ohla K., Veldhuizen M.G., Joseph P.V., Kelly C.E., Bakke A.J., Steele K.E., Farruggia M.C., Pellegrino R., Pepino M.Y. (2021). Recent smell loss is the best predictor of COVID-19 among individuals with recent respiratory symptoms. Chem. Senses.

[7] Boesveldt S., de Graaf K. (2017). The Differential Role of Smell and Taste For Eating Behavior. Perception.

[8] Roxbury C.R., Bernstein I.A., Lin S.Y., Rowan N.R. (2022). Association Between Chemosensory Dysfunction and Diet Quality in United States Adults. Am. J. Rhinol. Allergy.

[9] Abeywickrema S., Ginieis R., Oey I., Perry T., Keast R.S., Peng M. (2023). Taste but not smell sensitivities are linked to dietary macronutrient composition. Appetite.

[10] Ginieis R., Abeywickrema S., Oey I., Keast R.S., Peng M. (2022). Searching for individual multi-sensory fingerprints and their links with adiposity–New insights from meta-analyses and empirical data. Food Qual. Prefer..

[11] Romero-Gameros C.A., Waizel-Haiat S., Mendoza-Zubieta V., Anaya-Dyck A., López-Moreno M.A., Colin-Martinez T., Martínez-Ordaz J.L., Ferat-Osorio E., Vivar-Acevedo E., Vargas-Ortega G. (2020). Evaluation of predictive value of olfactory dysfunction, as a screening tool for COVID-19. Laryngoscope Investig. Otolaryngol..

[12] Bailey R.L., Mitchell D.C., Miller C., Smiciklas-Wright H. (2007). Assessing the Effect of Underreporting Energy Intake on Dietary Patterns and Weight Status. J. Am. Diet. Assoc..

[13] Saniasiaya J., Islam M.A., Abdullah B. (2021). Prevalence of Olfactory Dysfunction in Coronavirus Disease 2019 (COVID-19): A Meta-analysis of 27,492 Patients. Laryngoscope.

[14] Butowt R., Bilińska K., von Bartheld C. (2022). Why does the Omicron Variant Largely Spare Olfactory Function? Implications for the Pathogenesis of Anosmia in COVID-19. J. Infect. Dis..

[15] Boscolo-Rizzo P., Tirelli G., Meloni P., Hopkins C., Madeddu G., De Vito A., Gardenal N., Valentinotti R., Tofanelli M., Borsetto D. (2022). Coronavirus disease 2019 (COVID-19)–related smell and taste impairment with widespread diffusion of severe acute respiratory syndrome–coronavirus-2 (SARS-CoV-2) Omicron variant. Int. Forum Allergy Rhinol..

[16] McWilliams M.P., Coelho D.H., Reiter E.R., Costanzo R.M. (2022). Recovery from COVID-19 smell loss: Two-years of follow up. Am. J. Otolaryngol..

[17] Parker J.K., Methven L., Pellegrino R., Smith B.C., Gane S., Kelly C.E. (2022). Emerging Pattern of Post-COVID-19 Parosmia and Its Effect on Food Perception. Foods.

[18] Tan B.K.J., Han R., Zhao J.J., Tan N.K.W., Quah E.S.H., Tan C.J., Chan Y.H., Teo N.W.Y., Charn T.C., See A. (2022). Prognosis and persistence of smell and taste dysfunction in patients with COVID-19: Meta-analysis with parametric cure modelling of recovery curves. Bmj.

[19] Coelho D.H., Reiter E.R., Budd S.G., Shin Y., Kons Z.A., Costanzo R.M. (2021). Quality of life and safety impact of COVID-19 associated smell and taste disturbances. Am. J. Otolaryngol..

[20] Ammar A., Brach M., Trabelsi K., Chtourou H., Boukhris O., Masmoudi L., Bouaziz B., Bentlage E., How D., Ahmed M. (2020). Effects of COVID-19 Home Confinement on Eating Behaviour and Physical Activity: Results of the ECLB-COVID19 International Online Survey. Nutrients.

[21] Bennett G., Young E., Butler I., Coe S. (2021). The Impact of Lockdown During the COVID-19 Outbreak on Dietary Habits in Various Population Groups: A Scoping Review. Front. Nutr..

[22] Molina-Montes E., Uzhova I., Verardo V., Artacho R., García-Villanova B., Jesús Guerra-Hernández E., Kapsokefalou M., Malisova O., Vlassopoulos A., Katidi A. (2021). Impact of COVID-19 confinement on eating behaviours across 16 European countries: The COVIDiet cross-national study. Food Qual. Prefer..

[23] Buckland N.J., Kemps E. (2021). Low craving control predicts increased high energy density food intake during the COVID-19 lockdown: Result replicated in an Australian sample. Appetite.

[24] Buckland N.J., Swinnerton L.F., Ng K., Price M., Wilkinson L.L., Myers A., Dalton M. (2021). Susceptibility to increased high energy dense sweet and savoury food intake in response to the COVID-19 lockdown: The role of craving control and acceptance coping strategies. Appetite.

[25] Gallo L.A., Gallo T.F., Young S.L., Moritz K.M., Akison L.K. (2020). The Impact of Isolation Measures Due to COVID-19 on Energy Intake and Physical Activity Levels in Australian University Students. Nutrients.

[26] Di Renzo L., Gualtieri P., Pivari F., Soldati L., Attinà A., Cinelli G., Leggeri C., Caparello G., Barrea L., Scerbo F. (2020). Eating habits and lifestyle changes during COVID-19 lockdown: An Italian survey. J. Transl. Med..

[27] Oldham M., Garnett C., Brown J., Kale D., Shahab L., Herbec A. (2021). Characterising the patterns of and factors associated with increased alcohol consumption since COVID-19 in a UK sample. Drug Alcohol Rev..

[28] Neuman N., Sandvik P., Lindholm N.B., Bömer-Schulte K., Lövestam E. (2023). Food-related experiences and behavioral responses among people affected by chemosensory dysfunctions following COVID-19: A scoping review. Res. Nurs. Health.

[29] Chaaban N., Høier A.T.Z.B., Andersen B.V. (2021). A Detailed Characterisation of Appetite, Sensory Perceptional, and Eating-Behavioural Effects of COVID-19: Self-Reports from the Acute and Post-Acute Phase of Disease. Foods.

[30] Kaufman-Shriqui V., Navarro D.A., Raz O., Boaz M. (2022). Dietary changes and anxiety during the coronavirus pandemic: A multinational survey. Eur. J. Clin. Nutr..

[31] Mekanna A.N., Panchal S.K., Li L. (2022). Beyond lockdowns: A systematic review of the impacts of COVID-19 lockdowns on dietary pattern, physical activity, body weight, and food security. Nutr. Rev..

[32] Bertrand L., Shaw K.A., Ko J., Deprez D., Chilibeck P.D., Zello G.A. (2021). The impact of the coronavirus disease 2019 (COVID-19) pandemic on university students’ dietary intake, physical activity, and sedentary behaviour. Appl. Physiol. Nutr. Metab..

[33] Li Y. (2022). Dietary Intake of Young Adult College Students Before and During the COVID-19 Pandemic. Master’s Thesis.

[34] Baker M.G., Kvalsvig A., Verrall A.J. (2020). New Zealand’s COVID-19 elimination strategy. Med. J. Aust..

[35] Jefferies S., French N., Gilkison C., Graham G., Hope V., Marshall J., McElnay C., McNeill A., Muellner P., Paine S. (2020). COVID-19 in New Zealand and the impact of the national response: A descriptive epidemiological study. Lancet Public Health.

[36] Ministry of Health COVID-19: Minimisation and Protection Strategy for Aotearoa New Zealand. https://www.health.govt.nz/covid-19-novel-coronavirus/covid-19-response-planning/covid-19-minimisation-and-protection-strategy-aotearoa-new-zealand.

[37] Mathieu E., Ritchie H., Rodés-Guirao L., Appel C., Giattino C., Hasell J., Macdonald B., Dattani S., Beltekian D., Ortiz-Ospina E. (2020). Coronavirus Pandemic (COVID-19). https://ourworldindata.org/coronavirus.

[38] New Zealand Government Unite against COVID-19: Testing and Isolation. https://covid19.govt.nz/testing-and-isolation/if-you-have-covid-19/.

[39] Gerritsen S., Egli V., Roy R., Haszard J., Backer C.D., Teunissen L., Cuykx I., Decorte P., Pabian S.P., Van Royen K. (2021). Seven weeks of home-cooked meals: Changes to New Zealanders’ grocery shopping, cooking and eating during the COVID-19 lockdown. J. R. Soc. N. Z..

[40] Amataiti T.A., Hood F., Krebs J.D., Weatherall M., Hall R.M. (2021). The Impact of COVID-19 on diet and lifestyle behaviours for pregnant women with diabetes. Clin. Nutr. ESPEN.

[41] Stats NZ COVID-19 Data Portal. https://www.stats.govt.nz/experimental/covid-19-data-portal.

[42] Te Whatu Ora Health New Zealand COVID-19: Current Cases. https://www.tewhatuora.govt.nz/our-health-system/data-and-statistics/covid-19-data/covid-19-current-cases/#covid-19-by-location.

[43] Harris P.A., Taylor R., Thielke R., Payne J., Gonzalez N., Conde J.G. (2009). A metadata-driven methodology and workflow process for providing translational research informatics support. J. Biomed. Inf..

[44] Aschenbrenner K., Hummel C., Teszmer K., Krone F., Ishimaru T., Seo H.-S., Hummel T. (2008). The Influence of Olfactory Loss on Dietary Behaviors. Laryngoscope.

[45] Croy I., Nordin S., Hummel T. (2014). Olfactory Disorders and Quality of Life—An Updated Review. Chem. Senses.

[46] van Strien T., Frijters J.E.R., Bergers G.P.A., Defares P.B. (1986). The Dutch Eating Behavior Questionnaire (DEBQ) for assessment of restrained, emotional, and external eating behavior. Int. J. Eat. Disord..

[47] Biró G., Hulshof K., Ovesen L., Amorim Cruz J.A., for the E.G. (2002). Selection of methodology to assess food intake. Eur. J. Clin. Nutr..

[48] Dos Santos H., Halawani R., Jehi T., Khan R. (2023). Effect of COVID-19 outbreak on the diet, body weight and food security status of students of higher education: A systematic review. Br. J. Nutr..

[49] Brouillard A.M., Kraja A.T., Rich M.W. (2019). Trends in Dietary Sodium Intake in the United States and the Impact of USDA Guidelines: NHANES 1999-2016. Am. J. Med..

[50] Bolton K.A., Webster J., Dunford E.K., Jan S., Woodward M., Bolam B., Neal B., Trieu K., Reimers J., Nowson C. (2020). Sources of dietary sodium and implications for a statewide salt reduction initiative in Victoria, Australia. Br. J. Nutr..

[51] Tomasa-Irriguible T.-M., Bielsa-Berrocal L., Bordejé-Laguna L., Tural-Llàcher C., Barallat J., Manresa-Domínguez J.-M., Torán-Monserrat P. (2021). Low Levels of Few Micronutrients May Impact COVID-19 Disease Progression: An Observational Study on the First Wave. Metabolites.

[52] Voelkle M., Gregoriano C., Neyer P., Koch D., Kutz A., Bernasconi L., Conen A., Mueller B., Schuetz P. (2022). Prevalence of Micronutrient Deficiencies in Patients Hospitalized with COVID-19: An Observational Cohort Study. Nutrients.

[53] McAuliffe S., Ray S., Fallon E., Bradfield J., Eden T., Kohlmeier M. (2020). Dietary micronutrients in the wake of COVID-19: An appraisal of evidence with a focus on high-risk groups and preventative healthcare. BMJ Nutr. Prev. Health.

[54] Astrup A., Bügel S. (2010). Micronutrient deficiency in the aetiology of obesity. Int. J. Obes..

[55] Menni C., Valdes A.M., Polidori L., Antonelli M., Penamakuri S., Nogal A., Louca P., May A., Figueiredo J.C., Hu C. (2022). Symptom prevalence, duration, and risk of hospital admission in individuals infected with SARS-CoV-2 during periods of omicron and delta variant dominance: A prospective observational study from the ZOE COVID Study. Lancet.

[56] Wang J., Chen Y., Huang J., Niu C., Zhang P., Yuan K., Zhu X., Jin Q., Ran S., Huang Z. (2023). Prevalence of taste and smell dysfunction in mild and asymptomatic COVID-19 patients during Omicron prevalent period in Shanghai, China: A cross-sectional survey study. BMJ Open.

[57] Coelho D.H., Reiter E.R., French E., Costanzo R.M. (2023). Decreasing Incidence of Chemosensory Changes by COVID-19 Variant. Otolaryngol. Head Neck Surg..

[58] Abeywickrema S., Ginieis R., Oey I., Peng M. (2022). Olfactory and Gustatory Supra-Threshold Sensitivities Are Linked to Ad Libitum Snack Choice. Foods.

[59] González-Monroy C., Gómez-Gómez I., Olarte-Sánchez C.M., Motrico E. (2021). Eating Behaviour Changes during the COVID-19 Pandemic: A Systematic Review of Longitudinal Studies. Int. J. Environ. Res. Public Health.

[60] Bakaloudi D.R., Jeyakumar D.T., Jayawardena R., Chourdakis M. (2022). The impact of COVID-19 lockdown on snacking habits, fast-food and alcohol consumption: A systematic review of the evidence. Clin. Nutr..

[61] Dhakal C., Acharya B., Wang S. (2022). Food spending in the United States during the first year of the COVID-19 pandemic. Front. Public Health.

[62] Huber B.C., Steffen J., Schlichtiger J., Brunner S. (2021). Altered nutrition behavior during COVID-19 pandemic lockdown in young adults. Eur. J. Nutr..

[63] Sidebottom C., Ullevig S., Cheever K., Zhang T. (2021). Effects of COVID-19 pandemic and quarantine period on physical activity and dietary habits of college-aged students. Sports Med. Health Sci..

[64] Maganja D., Buckett K., Stevens C., Flynn E. (2019). Consumer choice and the role of front-of-pack labelling: The Health Star Rating system. Public Health Res. Pract..

[65] Abe-Inge V., Aidoo R., Moncada de la Fuente M., Kwofie E.M. (2024). Plant-based dietary shift: Current trends, barriers, and carriers. Trends Food Sci. Technol..

[66] Garden L., Clark H., Whybrow S., Stubbs R.J. (2018). Is misreporting of dietary intake by weighed food records or 24-hour recalls food specific?. Euro J. Clin. Nutr..

[67] Karvetti R.-L., Knuts L.-R. (1992). Validity of the estimated food diary: Comparison of 2-day recorded and observed food and nutrient intakes. J. Am. Diet. Assoc..

