# Supplementary material for: Impacts of COVID-19 on Food Choices and Eating Behavior among New Zealand University Students
Source: Foods. 2024 Mar 15;13(6):889. doi: 10.3390/foods13060889 (PMC10969711; doi:10.3390/foods13060889)
Supplement: Supplementary file 1 [file foods-13-00889-s001.zip › foods-2915521-supplementary.pdf]

Supplementary Materials S1: Survey Questions

#	Question	Responses
	**Section 1: About You**	
**1.01**	What year were you born?	
**1.02**	What is your gender?	Male<br>Female<br>Another gender<br>Prefer not to say
**1.03**	What is your ethnicity? (Check all that apply)	New Zealand European<br>Māori<br>Samoan<br>Cook Island Māori<br>Tongan<br>Niuean<br>Tokelauan<br>Fijian<br>Fijian Indian<br>Other Pacific Peoples<br>Chinese<br>Indian<br>Other Asian<br>Other: _____
**1.04a**	Is this your first year of tertiary study in Dunedin?	Yes<br>No
**1.04b**	What year are you in your tertiary studies?	First year<br>Second year<br>Third year / final year<br>Post-graduate
**1.04c**	What region did you live in before coming to study in Dunedin?	

**1.05**	Where were you located during the Level 4 lockdown of March/April 2020?	Dunedin<br>Auckland<br>Wellington<br>Other New Zealand city<br>Outside of New Zealand: ______
**1.05a**	Were you located in Auckland for any of the following periods? (select all that apply)	1, May 2020<br>2, August to September 2020<br>3, February to March 2021<br>4, August to November 2021<br>5, December 2021
**1.06a**	What best describes your current household/living situation?	Living alone<br>Living with other adults (e.g., flat or house share)<br>Married/de facto couple living alone (i.e., no children)<br>Family with children<br>Family with adults only<br>Single adult with children<br>Living in a Residential College<br>Other: ______________
**1.06b**	What best describes your household/living situation for most of 2019?	Living alone<br>Living with other adults (e.g., flat or house share)<br>Married/de facto couple living alone (i.e., no children)<br>Family with children<br>Family with adults only<br>Single adult with children<br>Living in a Residential College<br>Other: ______________

**1.07**	Are you taking any antidepressant or antianxiety medication?	Yes<br>No
**1.08**	Do you smoke cigarettes or vape (use e-cigarettes)?	1, Do not smoke or vape<br>2, Only smoke cigarettes occasionally<br>3, Only vape occasionally<br>4, Smoke cigarettes regularly (i.e., every day)<br>5, Vape regularly (i.e., every day)<br>6, Smoke cigarettes and vape occasionally<br>7, Smoke cigarettes and vape regularly (i.e., every day)

#	Question	Responses
	**Section 2: Experience of COVID-19**	
**2.01**	To your knowledge, how many times have you been infected with COVID-19 since February 2020?	Once<br>Twice<br>Three times<br>Four times<br>Five or more times
**2.02**	How was your COVID-19 infection confirmed? (select all that apply)	PCR test<br>RAT test<br>Positive household contact<br>Diagnosed by a medical professional<br>Infection not confirmed by a test
**2.02**	If known to you, please indicate which COVID-19 variant(s) you were infected with. (Select all that apply)	Original strain<br>Alpha<br>Beta<br>Gamma<br>Delta<br>Omicron<br>Other

		Don’t know
	The remaining questions will be about your **most recent** COVID-19 infection.	
**2.03**	Please indicate the month and year that you were most recently infected with COVID-19	MM/YY
**2.03a**	Where were you located at the time of infection?	Dunedin<br>Auckland<br>Wellington<br>Other New Zealand city<br>Outside of New Zealand
**2.03b**	Had you received any doses of the COVID-19 vaccine at the time of infection?	No<br>First dose<br>Second dose<br>Third primary dose<br>Booster
**2.04**	Which of the following symptoms did you experience during your COVID-19 infection? (select all that apply)	Sneezing and runny nose<br>Sore throat<br>Body aches<br>Fever<br>Cough<br>Tiredness<br>Headache<br>Nausea and vomiting<br>Diarrhea<br>Difficulty breathing<br>Chest pains<br>Other: __________
**2.05**	Overall, how severe were your symptoms of COVID-19?	Mild<br>Moderate<br>Severe

**2.06**	Did you experience any changes in smell or taste during your COVID-19 infection?	Yes<br>No
**2.06a**	What changes did you experience during your COVID-19 infection? (Please select all that apply)	Complete loss of smell<br>Reduced sense of smell<br>Complete loss of taste<br>Reduced sense of taste<br>Other sensory changes<br>Experienced with previous infection
**2.06b**	Please explain the changes you experienced, including their onset and any specific flavors or smells that you noticed a change in	(free text)
**2.06c**	How long did it take for your sense of smell or taste to return to normal?	Within 7 days of first symptoms<br>7 to 14 days after first symptoms<br>2 to 4 weeks<br>1 to 2 months<br>3 to 6 months<br>6 months or longer<br>Sensory changes have not returned to normal
**2.07a**	Have you experienced any change in the intensity of sweet foods (e.g., lollies, ice cream) since your most recent COVID-19 infection?	Tastes weaker<br>No change<br>Tastes stronger<br>Don’t know
**2.07b**	Have you experienced any change in the intensity of salty foods (e.g., ready-salted chips, pretzels) since your COVID-19 infection?	Tastes weaker<br>No change<br>Tastes stronger<br>Don’t know
**2.07c**	Have you experienced any change in the intensity of fatty foods (e.g., deep-fried foods) since your COVID-19 infection?	Tastes weaker<br>No change<br>Tastes stronger

		Don’t know
**2.08**	Compared to this time of year in 2019 (i.e., Pre-COVID), do you think you are eating more or less of the following:	
**2.08a**	Fast food (e.g., McDonalds)	Much more<br>Somewhat more<br>About the same<br>Somewhat less<br>Much less
**2.08b**	Restaurant meals	Much more<br>Somewhat more<br>About the same<br>Somewhat less<br>Much less
**2.08c**	Bakery food	Much more<br>Somewhat more<br>About the same<br>Somewhat less<br>Much less
**2.08d**	Meal box subscriptions	Much more<br>Somewhat more<br>About the same<br>Somewhat less<br>Much less
**2.08e**	Homemade baked goods	Much more<br>Somewhat more<br>About the same<br>Somewhat less<br>Much less
**2.08f**	Meals prepared at home from scratch	Much more<br>Somewhat more

		About the same<br>Somewhat less<br>Much less
**2.08g**	Savory snack foods (e.g., crackers, chips)	Much more<br>Somewhat more<br>About the same<br>Somewhat less<br>Much less
**2.08h**	Sweet snack foods (e.g., chocolate, dried fruit)	Much more<br>Somewhat more<br>About the same<br>Somewhat less<br>Much less
**2.08i**	Soft drinks	Much more<br>Somewhat more<br>About the same<br>Somewhat less<br>Much less
**2.08j**	Alcohol	Much more<br>Somewhat more<br>About the same<br>Somewhat less<br>Much less
**2.09**	Are there any changes that you have made to how or what you eat since the start of the COVID-19 pandemic?	(free text)
**2.10**	What impacts, if any, do you believe COVID-19 has had on how or what you eat?	(free text)

**#**	**Question**	**Responses**
	**Section 3: Eating Behaviour**	

**3.34**	Do you follow a specific diet (i.e., plant-based, Mediterranean, keto, paleo, religious regulation)?	Yes<br>No
**3.34a**	What diet do you follow?	Plant-based diet (including vegan and vegetarian diets)<br>Mediterranean diet<br>Low-carb diet (including Ketogenic diet, Atkins)<br>Low-carbon diet<br>Religious dietary regulations (such as Kosher or Halal foods)<br>Other: __________
**3.35**	Do you have any allergies or intolerance or health conditions that affect your diet?	No<br>Yes: ______________
**3.36**	What best describes your typical meal preparation?	Meal is usually prepared by someone else (i.e., residential college, parents, flatmate)<br>Usually prepared the meal myself from scratch<br>Usually buy pre-prepared meals (i.e., takeaways)<br>Other: __________
**3.37**	How much do you typically spend on food overall for yourself in a normal week?	Under $50<br>$50 to $99.99<br>$100 to $150<br>$150 to $200<br>$200 to $300<br>More than $300
**3.38**	How much do you typically spend on takeaway food in a normal week?	Under $25<br>$25 to $49.99<br>$50 to $75<br>$75 to $100<br>More than $100
**3.39**	In general, would you say your health is:	Excellent<br>Very good<br>Good<br>Fair<br>Poor<br>Don’t know
